# Choroidal structural evaluation in celiac disease

**DOI:** 10.1038/s41598-021-95812-y

**Published:** 2021-08-12

**Authors:** Maddalena De Bernardo, Livio Vitiello, Michela Battipaglia, Francesca Mascolo, Claudio Iovino, Luigi Capasso, Carolina Ciacci, Nicola Rosa

**Affiliations:** 1 Eye Unit, Department of Medicine, Surgery and Dentistry, “Scuola Medica Salernitana”, University of Salerno, Baronissi, Salerno Italy; 2 Celiac Centre at University Hospital San Giovanni Di Dio e Ruggi d’Aragona, University of Salerno, Baronissi, Salerno Italy; 3 Eye Clinic, Multidisciplinary Department of Medical, Surgical and Dental Sciences, University of Campania Luigi Vanvitelli, Naples, Italy; 4 Corneal Transplant Unit, ASL Napoli 1, Naples, Italy

**Keywords:** Coeliac disease, Eye diseases

## Abstract

This observational case–control study assessed the differences in choroidal structure between patients with celiac disease and healthy subjects utilizing the choroidal vascularity index (CVI). Seventy-four celiac patients and 67 healthy subjects underwent a complete ophthalmological evaluation, axial length (AL) measurements and spectral-domain optical coherence tomography with enhanced depth imaging mode (EDI SD-OCT) evaluation. These images were binarized and choroidal vasculature was analyzed. Choroidal total subfoveal area (TSA), luminal subfoveal area (LSA), stromal subfoveal area (SSA), CVI and subfoveal choroidal thickness (CT) were measured. Furthermore, subfoveal CT, TSA, LSA, SSA, and CVI were also correlated with AL. A statistically significant difference was found between the two groups for TSA, LSA, SSA and subfoveal CT, but not for CVI. In celiac patients, a significant correlation was found between AL and TSA, LSA and SSA, but not with CVI. Similar findings were also noticed in the healthy subjects. Thus, celiac patients have a thicker choroid than healthy subjects, regardless of the AL, due to a proportional increase in both the vascular and stromal components, which does not alter the CVI.

## Introduction

Celiac disease is an autoimmune inflammatory disorder mostly affecting the proximal small intestine. It is caused by autoantibodies produced against tissue transglutaminase that are activated in genetically predisposed people by gluten and gluten-like proteins^1^. Once considered an intestine-related disorder, celiac disease is now known as a systemic, chronic and immune-mediated condition^2^. In particular, the autoantibodies identified in celiac disease bind to different extraintestinal tissues and cause a variety of immunological diseases, such as osteopenia, dermatitis herpetiformis, iron deficiency anemia, dilated cardiomyopathy, myocarditis, peripheral neuropathy, ataxia, epilepsy, liver diseases, and glomerulonephritis^3^.

Some case reports have shown that celiac disease may involve the eye with different clinical presentations^4–7^.

In fact, Al Hemidan et al.^4^ discussed a case of Vogt-Koyanagi-Harada disease in a diabetic 3-year-old girl with celiac disease, Krifa et al.^5^ dealt with a diabetic and celiac 9-year-old girl presenting with uveitis responding to a gluten-free diet, while Malhi et al.^6^ reported two cases of central retinal vein occlusion in two Indian girls with an unknown diagnosis of celiac disease.

Hence, ocular involvement in celiac disease may include immunogenetic factors, cell antigenic epitopes cross-reactivity, immune complexes or autoantibodies increase, and several vitamins deficiencies^4^. Furthermore, particular attention should be paid to the vascular component of the eye, as the clinical manifestations previously discussed demonstrate a predominant involvement of this anatomical area.

The choroid is a densely vascularized structure that contributes to the greatest supply of oxygen and other nutrients to the outer retina and the retinal pigment epithelium^8^. It is one of the highest blood flow body tissues, and most of its anatomical knowledge derives from postmortem histological studies^9,10^.

Over the last decades, optical coherence tomography (OCT) has permitted thorough and non-invasive in vivo analysis of the retina and choroid. More recent technical developments with the introduction of spectral-domain optical coherence tomography with enhanced depth imaging mode (EDI SD-OCT) evaluation, swept-source OCT, and OCT angiography have allowed a more comprehensive study of the choroidal structure. Although choroidal thickness (CT) measurement is considered a reliable tool in clinical research, it represents the whole choroidal structure, with no distinction between the luminal and stromal components^11–13^.

The choroidal vascularity index (CVI) is becoming a new imaging method for choroidal vascular system measurement and analysis by quantifying both choroidal stromal and luminal parts^14,15^. CVI shows less variability than CT and it is affected by fewer physiological factors, so it is considered a relatively stable parameter to assess changes in choroidal vasculature^14^.

To the best of our knowledge, there has been limited investigation of choroidal vasculature in patients with celiac disease^16–18^, all of which have provided conflicting results, and none of these previous studies evaluated the CVI.

The purpose of this study is to evaluate the differences in choroidal structure between celiac patients and healthy subjects with CVI, trying to better understand the underlying pathogenetic mechanism of celiac disease in ocular manifestations.

## Materials and methods

### Patients selection

In this observational case–control study, adult subjects with a diagnosis of celiac disease, consecutively seen at the Celiac Disease Center, and a control group of normal subjects, recruited among spouses of patients and hospital staff, were enrolled between September 2019 and March 2020 at the Department of Medicine, Surgery and Dentistry of the University of Salerno.

Patients with a diagnosis of celiac disease confirmed by serology and intestinal biopsy, regardless of the time of diagnosis, were included. Controls were chosen among persons with no diagnosis of gastrointestinal diseases, and at least one negative celiac disease-specific serology.

Subjects with systemic and ocular diseases, other than celiac disease, which could affect choroidal vasculature, subjects presenting clinical features compatible with a diagnosis of glaucoma, patients younger than 18 or older than 70 years of age, eyes with an axial length (AL) less than 21 mm or more than 26.5 mm and pregnant women were excluded. Furthermore, amblyopic eyes and eyes with a best corrected visual acuity less than 20/25 were also excluded.

The established refraction limits were set < 5D (spherical) and < 3D (cylindrical) refractive error.

The present study adheres to the ethical principles of the Declaration of Helsinki.

All the participants were carefully informed about the purpose of the study and written informed consent from each subject was acquired. Institutional Review Board (IRB) approval was also obtained from the ComEtico Campania Sud (CECS), prot. n°16544.

### Clinical and instrumental examination

Patients underwent a complete ophthalmological evaluation, including clinical history, Snellen best corrected visual acuity, AL measurements with IOLMaster (Carl Zeiss Meditec AG, Jena, Germany, version 5.4.4.0006) and EDI SD-OCT evaluation (Spectralis; Heidelberg Engineering; Heidelberg, Germany, version 6.0).

**Figure 1:**
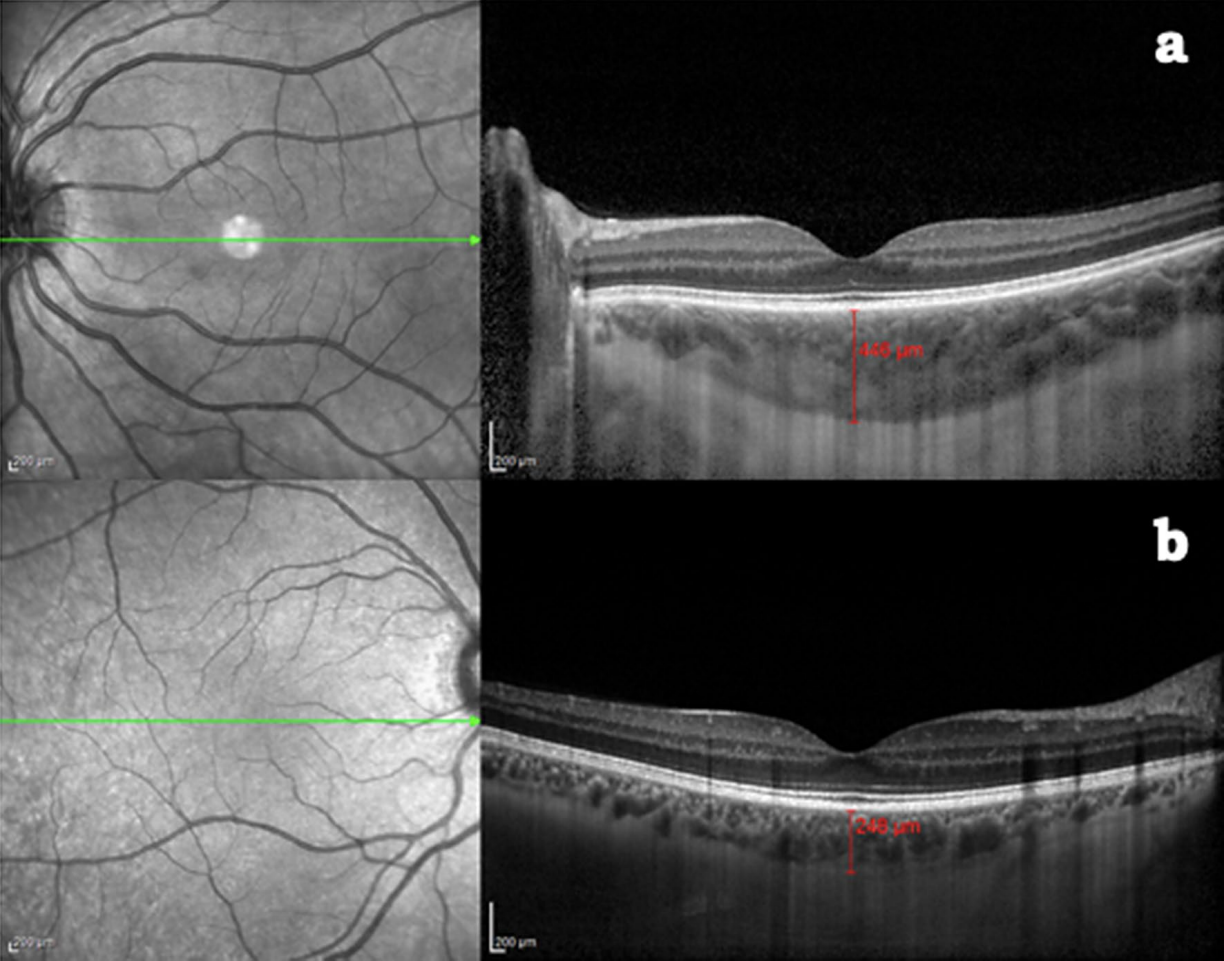
Choroidal thickness measured with the built-in software of the device at the subfoveal level in: (**a**) celiac patient. (**b**) Healthy volunteer.

**Figure 2:**
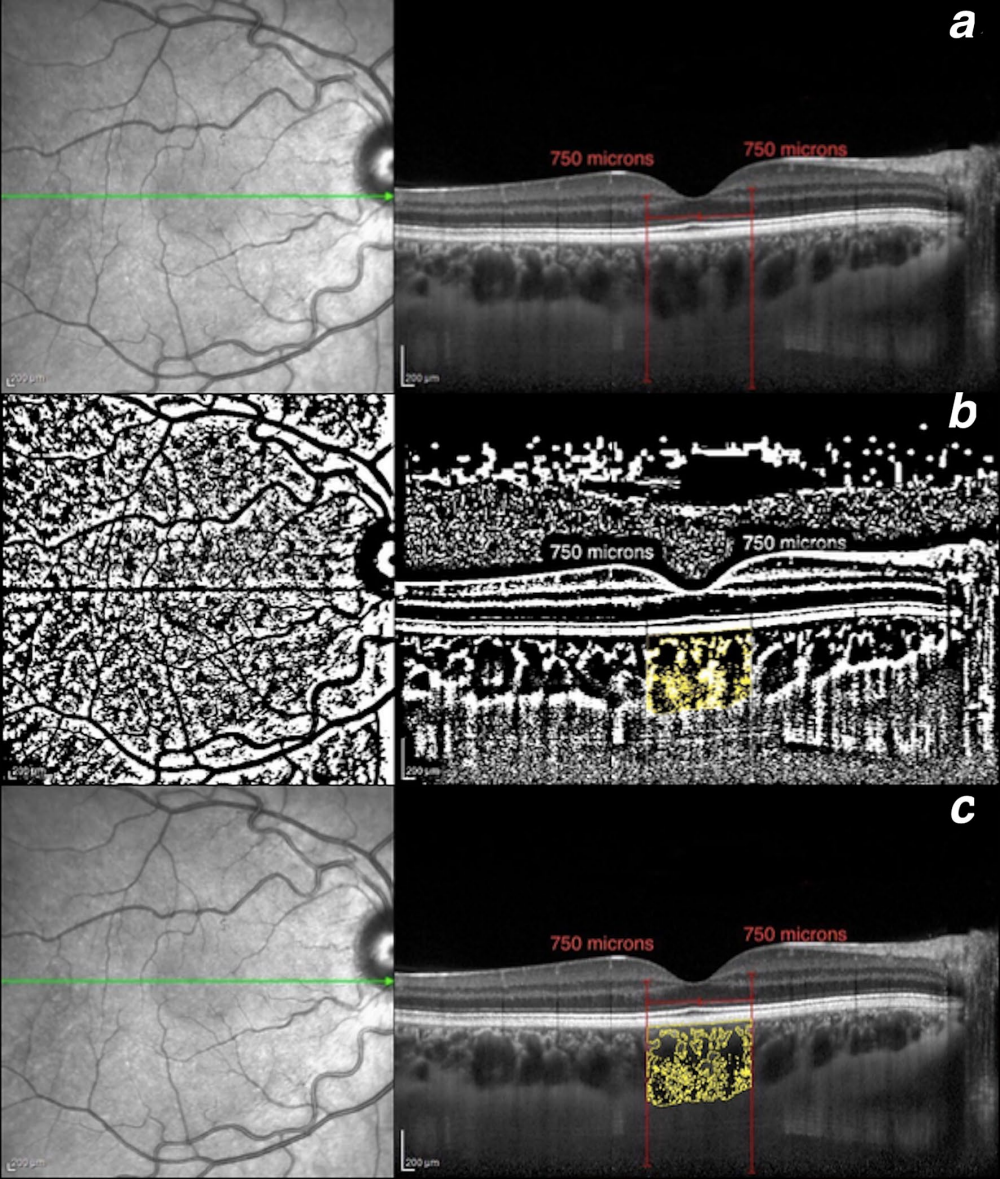
Choroidal vascularity index calculation in a healthy volunteer. (**a**) Determination of the subfoveal choroidal region in the original EDI SD-OCT image with the built-in software of the device. (**b**) Identification of the subfoveal luminal and stromal areas using the binarization technique. (**c**) Overlay of the target choroidal region created by image binarization on the EDI SD-OCT image.

All participants were examined between 2:00 p.m. and 3:00 p.m., reducing the bias related to the diurnal variations of choroidal thickness^19^, and without pupil dilation. A horizontal 30° linear OCT B-scan passing through the fovea was obtained for all examined eyes. Using the built-in software of the device (Heidelberg Eye Explorer HEYEX; Heidelberg Engineering), the subfoveal CT was measured as the distance between Bruch’s membrane and the sclera-choroidal interface under the fovea (Fig. 1a,b). Furthermore, a distance of 750 μm was manually measured nasally and temporally from the centre of the fovea on the OCT B-scan, parallel to the retinal pigment epithelium. Subsequently, two lines perpendicular to those previously drawn were manually prolonged until the sclerochoroidal junction, delimiting a subfoveal area (Fig. 2a).

For each patient, the eye with the best OCT quality image index was chosen to perform this evaluation. Choroidal total subfoveal area (TSA), choroidal luminal subfoveal area (LSA), choroidal stromal subfoveal area (SSA) and CVI were evaluated in both celiac and healthy subjects.

Low quality OCT images, due to optic media opacities, with an OCT imaging quality index < 20 decibels were excluded.

### OCT analysis: choroidal vascularity index (CVI)

All collected and analyzed images, together with subfoveal CT measurements, were reviewed by an expert in OCT evaluation (MDB).

In all eyes, macular B scan images were exported with a 1:1 pixel ratio and processed with ImageJ software (National Institutes of Health, Bethesda, USA, version 1.52q) using the set scale tool to convert pixels to μm. The TSA, LSA, SSA and CVI were measured and calculated by an examiner who was blind to the patient’s status, as suggested^20–22^.

Briefly, the polygon tool was used to select the previously delimitated choroidal subfoveal area in the OCT B-scan. After converting the image into 8 bit, Niblack’s auto-local threshold was applied to binarize the image and demarcate the LSA and SSA. LSA was highlighted by applying the color threshold and then added to the region of interest manager. The TSA and LSA were measured and the CVI (defined as the ratio of LSA to TSA) was calculated in each eye (Fig. 2b,c). SSA was obtained by subtracting LSA from TSA.

### Statistical analysis

All data were analyzed with SPSS Software (IBM SPSS Statistics version 25). For each parameter the mean, standard deviation, variance, standard error of the mean, variation coefficient, and minimum and maximum values were calculated. The Kolmogorov–Smirnov test showed a normal distribution (p > 0.05) for all data, except for age and SSA. In addition, one way analysis of variance was performed, showing similar variances between the two groups for AL and age. For this reason, to compare the different parameters of the two groups, the two-tailed independent samples Student’s *t*-test was used for normal-distributed data, while the two-tailed Mann–Whitney U test was used for age and SSA. Moreover, the Chi-Square test with Yates correction to assess potential differences for sex and myopia prevalence between the two groups was also performed. The Pearson correlation coefficient (r) was utilized to evaluate the correlation between AL and CT, the different choroidal areas and CVI of the two study groups, except for SSA of the celiac group, where the Spearman correlation coefficient (ρ) was used.

P values less than 0.05 were considered statistically significant.

The sample size was determined by maximizing the statistical power. The analysis was performed by using G*Power software (version 3.1.9.4)^23^. A difference between two independent means (two groups) was computed. Input data were the following: α was set at 0.05; 1-β was set at 0.838; allocation ratio N2/N1 was set at 0.91; effect size was set as medium at around 0.5. Results were the following: non-centrality parameter δ = 2.965; critical t = 1.977; Df = 139; sample size group 1 = 74; sample size group 2 = 67; actual power = 0.835; total sample size = 141.

## Results

A total of 74 celiac patients and 67 healthy volunteers, chosen among spouses of patients and hospital staff, were enrolled. Three celiac patients and with low quality OCT images were excluded. Sixty-eight patients out of 74 were following a gluten-free diet since the diagnosis of celiac disease (> 1 year). Six patients were examined after a few days from the diagnosis and the beginning of the gluten-free diet. The mean disease duration of patients with celiac disease was 9.2 ± 8.6 years (range: 0–41 years).

All clinical and choroidal characteristics of the two groups are summarized in Table 1. There were no statistically significant differences for age, AL and sex between the groups (p = 0.947, p = 0.119, and p = 0.065 respectively).

**Table 1:** Clinical and Choroidal Characteristics of the two study groups.

	Mean	SD	σ^2^	SEM	σ* (%)	Min	Max	p-value
**Age**								
Celiac group	40.78	11.33	128.37	1.32	27.78	18.00	66.00	0.947^a^
Control group	40.67	13.82	190.99	1.69	33.98	23.00	69.00	
**AL (mm)**								
Celiac group	23.59	0.95	0.90	0.11	4.04	21.70	26.12	0.119^b^
Control group	23.85	1.00	1.00	0.12	4.19	21.50	26.36	
**Sex (M/F)**								
Celiac group	21/53							0.065^c^
Control group	30/37							
**Subfoveal CT (µm)**								
Celiac group	372.38	92.82	8615.55	10.79	24.93	178.00	560.00	0.0002^b^
Control group	315.51	86.63	7504.76	10.58	27.46	74.00	532.00	
**TSA (mm**^**2**^**)**								
Celiac group	1.94	0.50	0.25	0.06	25.74	0.83	2.74	< 0.0001^b^
Control group	1.62	0.41	0.17	0.05	25.62	0.36	2.65	
**LSA (mm**^**2**^**)**								
Celiac group	1.31	0.34	0.12	0.04	25.88	0.55	2.03	< 0.0001^b^
Control group	1.09	0.27	0.07	0.03	24.63	0.26	1.66	
**SSA (mm**^**2**^**)**								
Celiac group	0.63	0.18	0.03	0.02	28.91	0.28	1.02	0.002^a^
Control group	0.53	0.17	0.03	0.02	32.16	0.10	1.09	
**CVI (%)**								
Celiac group	67.75	3.41	11.63	0.40	5.03	60.18	78.25	0.760^b^
Control group	67.55	4.16	17.31	0.51	6.16	58.83	76.74	

*SD* standard deviation, σ^2^ variance, *SEM* standard error of the mean, σ* variation coefficient, *AL* axial length, *CT* choroidal thickness, *TSA* total subfoveal area, *LSA* luminal subfoveal area, *SSA* stromal subfoveal area, *CVI* choroidal vascularity index.

^a^Mann Whitney U test.

^b^Student t-test unpaired.

^c^Chi-square test with Yates correction.

Concerning the prevalence of myopic eyes (AL > 24.5 mm) in the two study groups, (16.2% in the celiac group and 23.9% in the control group), no statistical significant difference was found (p = 0.354) .

Regarding subfoveal CT, a statistically significant difference was found between the two studied groups (p = 0.0002) (Table 1).

Concerning different subfoveal choroidal areas and CVI, a statistically significant difference was found between the two groups for TSA, LSA and SSA (p < 0.0001, p < 0.0001, p = 0.002, respectively), but not for CVI (p = 0.760) (Table 1).

Concerning the correlation between AL and the different choroidal parameters, for the celiac group a significant correlation was found between AL and all the choroidal parameters, except for CVI (Table 2).

**Table 2:** Correlation between AL and the choroidal parameters in the two study groups.

	r	95% CI	R^2^	p-value
Celiac group (74 patients)				
AL-CT	− 0.31	− 0.51 to − 0.09	0.10	0.007^a^
AL-TSA	− 0.37	− 0.55 to − 0.16	0.14	0.001^a^
AL-LSA	− 0.36	− 0.54 to − 0.14	0.13	0.002^a^
AL-SSA	− 0.32	− 0.51 to − 0.09	–	0.006^b^
AL-CVI	+ 0.03	− 0.20 to + 0.25	0.00062	0.833^a^
Healthy group (67 patients)				
AL-CT	− 0.35	− 0.54 to − 0.12	0.12	0.004^a^
AL-TSA	− 0.37	− 0.56 to − 0.14	0.13	0.002^a^
AL-LSA	− 0.37	− 0.56 to − 0.14	0.13	0.002^a^
AL-SSA	− 0.32	− 0.52 to − 0.08	0.10	0.009^a^
AL-CVI	+ 0.06	− 0.18 to + 0.30	0.0035	0.635^a^

*r* correlation coefficient, *95% CI* 95% confidence interval, *R*^*2*^ determination coefficient, *AL* axial length, *CT* choroidal thickness, *TSA* total subfoveal area, *LSA* luminal subfoveal area, *SSA* stromal subfoveal area, *CVI* choroidal vascularity index.

^a^Pearson correlation.

^b^Spearman correlation.

Regarding the control group, similar findings were noticed (Table 2).

**Figure 3:**
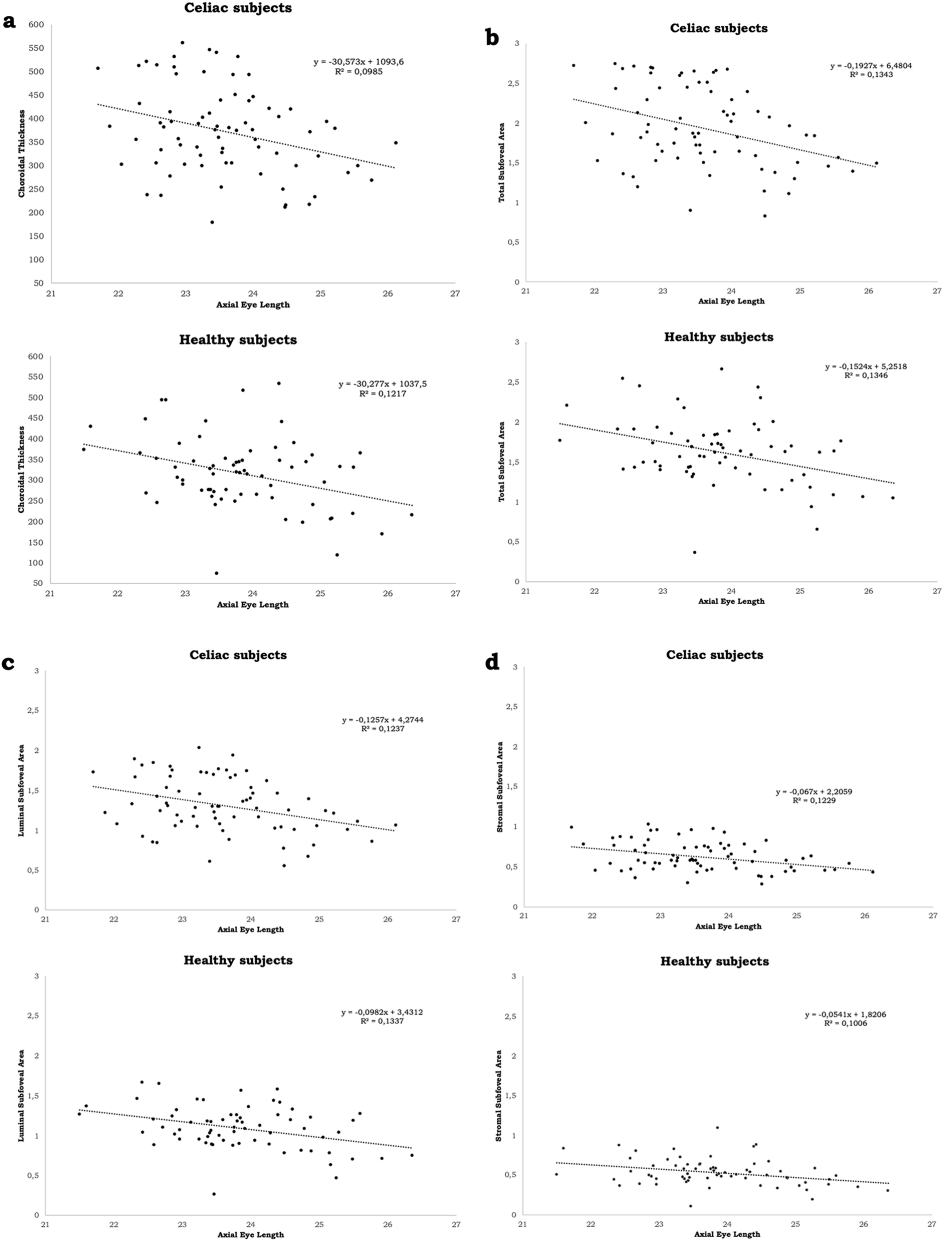

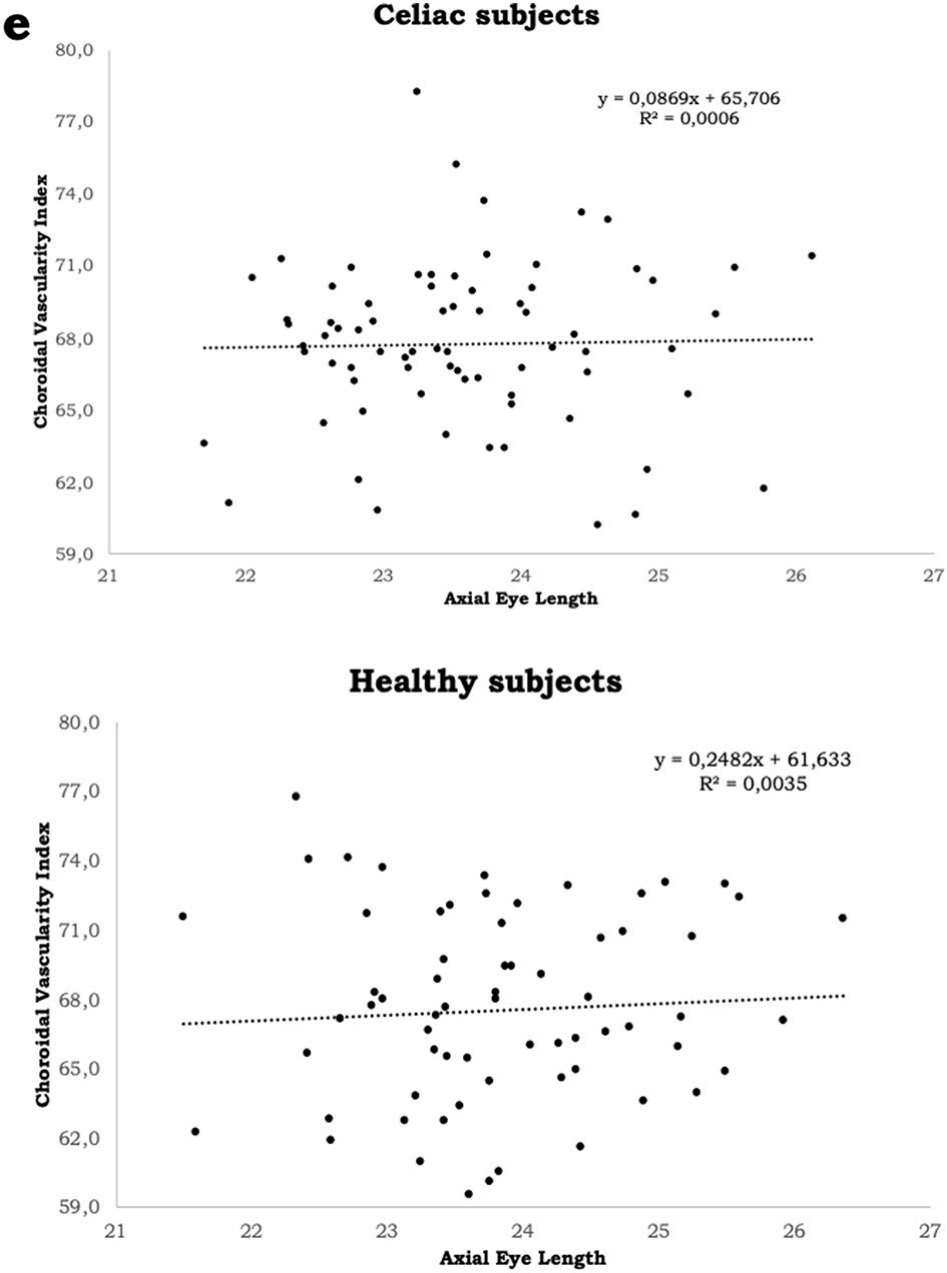
Scatter plot correlating axial length in the two study groups with: (**a**) subfoveal choroidal thickness (**b**) total subfoveal area. (**c**) Luminal subfoveal area. (**d**) Stromal subfoveal area. (**e**) Choroidal vascularity index. R^2^: determination coefficient. ImageJ: http://imagej.nih.gov/ij. GPower: https://www.psychologie.hhu.de/arbeitsgruppen/allgemeine-psychologie-und-arbeitspsychologie/gpower.html.

All the correlation graphs are depicted in the Fig. 3a–e.

No significant correlation between disease duration and choroidal parameters was found.

## Discussion

Celiac disease is an autoimmune disease with systemic involvement that mainly affects the small intestine, but it also presents several extraintestinal manifestations^3^. Among these, the eye certainly represents one of the target organs of the disease, and night blindness, dry eye, cataract, thyroid-associated orbitopathy, uveitis, central retinal vein occlusion and neuro-ophthalmic manifestations may occur^24^.

In this context, an in vivo study of the choroidal characteristics would provide interesting information.

CVI is a recent imaging tool that allows a more accurate evaluation of the choroidal structure, facilitating discrimination of the vascular and stromal components, and also providing their quantification^14,15^. This new parameter has already been used to evaluate different ocular and systemic disorders and pathological conditions^25–28^. To the best of our knowledge, this is the first study which utilizes CVI in the choroid appraisal and analysis of patients with celiac disease.

In addition to the increased subfoveal CT in celiac patients, the present study also showed that all subfoveal choroidal areas were thicker in these patients than in healthy controls, confirming the findings by Bolukbasi et al.^16^. Nevertheless, no statistical significance was found for CVI between the two studied groups.

Since CVI is the ratio of LSA to TSA, the absence of a statistically significant difference between the two groups could be explained by a proportional increase in all choroidal areas of celiac patients. This may be due to the simultaneous presence of inflammatory and autoimmune mechanisms, typical of celiac disease, which determine an increase of all the choroidal components, as already hypothesized in previous studies^16–18^. In fact, LSA may be enlarged by the increase of choroidal vessels due to inflammatory response in celiac disease, while SSA may be increased by the deposition of autoimmune complex in the choroidal stromal tissue. Consequently, the combined increase in LSA and SSA may lead to an increase in TSA.

Furthermore, in the present study, no significant correlation between disease duration and choroidal parameters was found. Probably, the thickness of the subfoveal choroidal areas is related to an immune-mediated alteration, that is not responsive to the gluten-free diet. A similar condition is also known for other abnormalities found in patients with celiac disease on a gluten-free diet, such as thyroiditis^29^, or insulin-dependent diabetes^30^. This could be related to irreversible changes caused by the disease itself, which are not affected by the the gluten-free diet. However, further studies in naive patients would be needed to better understand the choroidal baseline status of such subjects, and how it modifies over time.

In the literature, a few studies have evaluated the CT in celiac patients^16–18^, but none have evaluated the CVI.

Karatepe Hashas et al.^17^ evaluated 31 celiac children and 34 controls, performing a SD-OCT examination including macular thickness, retinal nerve fiber layer measurements, ganglion cell thickness, and choroidal measurements. In detail, in all studied participants CT was measured at 9 points: at subfovea, and 500 μm, 750 μm, 1000 μm, and 1500 μm nasal and temporal to the fovea. However, a statistically significant difference was found between the celiac group and the control group only for the retinal nerve fiber layer, showing a thinner one in celiac patients. The authors explained this finding by presuming an underlying autoimmune mechanism related to autoantibodies with an affinity to retinal nerve tissue.

Bolukbasi et al.^16^ carried out a study on 70 eyes of 70 patients with celiac disease, comparing them to 70 eyes of 70 healthy patients. The authors measured AL and performed an EDI SD-OCT evaluation of CT at 7 different points: at subfovea, and 500 μm, 1000 μm, and 1500 μm nasal and temporal to the fovea. They found no significant differences for AL between the two study groups but, contrarily to Karatepe Hashas et al.^17^, they found the choroid to be thicker at all predefined measurement points in celiac patients (all p < 0.001). No correlation between AL and CT was tested. The authors speculated that systemic inflammation in celiac disease causes the enlargement of choroidal vessels with a subsequent increase in CT.

In a case–control study, Doğan et al.^18^ analyzed 42 celiac children and 42 age and sex-matched healthy controls, correlating subfoveal CT measured with EDI SD-OCT to titers of anti-endomysial antibodies and tissue transglutaminase type 2 antibody, which are used in the diagnosis of celiac disease and following adherence to diet. The authors found that the mean CT of celiac patients was slightly thicker than those of healthy controls, but without any statistical significance. Furthermore, they found that patients with celiac disease who were non-adherent to a gluten-free diet had a thinner mean subfoveal CT than celiac patients adherent to a gluten-free diet. They tried to explain this finding speculating that circulating antibodies may have had a role in thinning the choroidal layers. Unfortunately, they were unable to prove circulating antibodies within choroidal microcirculation. Moreover, their data had no sufficient statistical significance, possibly due to the small sample size of the study, as the authors themselves stated.

Several papers have been published on the correlation between AL and CT in emmetropic and myopic patients, most of them showing AL to be inversely related to subfoveal CT^20,31–35^.

Sonoda et al.^20^ examined a large (7500-μm width) choroidal area of 180 right eyes of 180 healthy volunteers with an AL range between 21.9 and 27.7 mm, showing no statistically significant correlation between AL and total choroidal area, luminal area and stromal area. However, the authors found an inverse correlation between AL and the ratio of the luminal to stromal area, thus considering the luminal area to be more affected by reduction than the stromal area.

A different structure is hypothesized to be present in long eyes, as reported by Alshareef et al.^36^, who evaluated 30 eyes with myopia (AL > 25 mm) in comparison to age-matched healthy subjects. The authors demonstrated statistically significant differences between myopic and healthy eyes in total choroidal area and stromal area (p < 0.001) but, interestingly, they found no significant differences in vascular area or vessel-to-stroma ratio between the groups. They considered these differences among the two examined groups attributable to stromal area modifications rather than to vascular changes.

The present study is the first to evaluate and to correlate the choroidal parameters with AL in patients with and without celiac disease. In both groups, a statistically significant inverse correlation between AL and subfoveal CT, TSA, LSA and SSA, but not for CVI, was found.

This study has some limitations. First, subfoveal CT measurements and segmentation method were manually performed. However, we attempted to reduce this bias, as all the choroidal evaluations were performed by a well-trained masked examiner. Moreover, macula morphometry could vary with AL, so slightly different choroidal areas between subjects could have been analyzed.

In addition, this is a monocentric study, so different ethnicities and subjects from larger geographic areas could have enriched the study findings.

In conclusion, patients with celiac disease showed a thicker choroid than healthy patients, due to a proportional increase in both the vascular and stromal components, which did not change the CVI. This may be linked to the inflammatory and autoimmune responses related to celiac disease pathophysiology. Nevertheless, further validations are needed.

## Data Availability

The datasets generated and analyzed during the current study are available from the corresponding author on reasonable request.
